# Resting-state prefrontal EEG biomarker in correlation with postoperative delirium in elderly patients

**DOI:** 10.3389/fnagi.2023.1224264

**Published:** 2023-09-25

**Authors:** Jeongmin Kim, Sujung Park, Keung-Nyun Kim, Yoon Ha, Sang-Jun Shin, Wonseok Cha, Ki-young Lee, Jungmi Choi, Bon-Nyeo Koo

**Affiliations:** ^1^Department of Anesthesiology and Pain Medicine, Yonsei University College of Medicine, Seoul, Republic of Korea; ^2^Anesthesia and Pain Research Institute, Yonsei University College of Medicine, Seoul, Republic of Korea; ^3^Department of Neurosurgery, Spine and Spinal Cord Institute, Severance Hospital, Yonsei University College of Medicine, Seoul, Republic of Korea; ^4^POSTECH Biotech Center, Pohang University of Science and Technology, Pohang, Republic of Korea; ^5^Department of Biomedical Systems Informatics, Biostatistics Collaboration Unit, Yonsei University College of Medicine, Seoul, Republic of Korea; ^6^Human Anti-Aging Standards Research Institute, Gyeongsangnam-Do, Republic of Korea

**Keywords:** electroencephalography, elderly, median dominant frequency, postoperative delirium, postoperative complications

## Abstract

Postoperative delirium (POD) is associated with adverse outcomes in elderly patients after surgery. Electroencephalography (EEG) can be used to develop a potential biomarker for degenerative cerebral dysfunctions, including mild cognitive impairment and dementia. This study aimed to explore the relationship between preoperative EEG and POD. We included 257 patients aged >70 years who underwent spinal surgery. We measured the median dominant frequency (MDF), which is a resting-state EEG biomarker involving intrinsic alpha oscillations that reflect an idle cortical state, from the prefrontal regions. Additionally, the mini-mental state examination and Montreal cognitive assessment (MoCA) were performed before surgery as well as 5 days after surgery. For long-term cognitive function follow up, the telephone interview for cognitive status™ (TICS) was performed 1 month and 1 year after surgery. Fifty-two (20.2%) patients were diagnosed with POD. A multivariable logistic regression analysis that included age, MoCA score, Charlson comorbidity index score, Mini Nutritional Assessment, and the MDF as variables revealed that the MDF had a significant odds ratio of 0.48 (95% confidence interval 0.27–0.85). Among the patients with POD, the postoperative neurocognitive disorders could last up to 1 year. Low MDF on preoperative EEG was associated with POD in elderly patients undergoing surgery. EEG could be a novel potential tool for identifying patients at a high risk of POD.

## 1. Introduction

Patients undergoing surgery that requires anesthesia are at risk of developing perioperative cognitive impairment, including delirium (Hughes et al., 2020). Specifically, postoperative delirium (POD) in elderly patients can interfere with their postoperative return to daily life (Marcantonio, 2017). POD is associated with re-admission, long-term functional decline, increased duration of hospital stay, and adverse outcomes (Hernandez et al., 2017). The most common cognitive function test for delirium is the confusion assessment method (CAM), which screens for the following four features: (a) an acute onset and fluctuating course of mental state, (b) inattention, (c) disorganized thinking, and (d) altered level of consciousness (Ely et al., 2001). Since continuous close observation and interviews are necessary for POD diagnosis, trained personnel are required to evaluate the neurologic symptoms of patients.

Electroencephalography (EEG) is an electrophysiological tool for monitoring electrical activity with electrodes placed on the scalp. It is a non-invasive, cheap, and reproducible technique for measuring neural activity with good temporal resolution. Unlike neuropsychological assessments, EEG parameters are not influenced by the cultural background or education level (Polich, 2005). Numerous studies have described the diagnostic utility of EEG for cognitive dysfunction using parameters such as spectral measures and synchronization between brain regions (Babiloni et al., 2016). Patients with Alzheimer’s disease (AD) or mild cognitive impairment (MCI) usually present with slow oscillatory brain activity, decreased EEG complexity, synchrony, and coherence (Stam, 2010). Given the recent developments in brain-computer interfaces, EEG devices have become more practical in the form of wearable devices, including headsets or hairbands where sensors are attached to hairless areas of the frontal lobe without electrode paste (Kafashan et al., 2021). These single, wireless prefrontal electrode systems, which have improved usability and portability while maintaining data quality (Rogers et al., 2016), have facilitated the use of EEG in daily clinical practice. We reported the correlation of resting-state EEG slowing (measured using a two-channel prefrontal EEG system) with the mini-mental state exam (MMSE) global and cognitive domain scores, especially those for orientation to time and place (Choi et al., 2019). Additionally, using the same two-channel prefrontal EEG system, we reported that slow intrinsic oscillation is associated with mild cognitive impairment due to Alzheimer’s disease (Choi et al., 2023). This indicated that our two-channel pre-frontal EEG system could yield reliable data quality and be clinically useful to examine cognitive function (Choi et al., 2019, 2020; Yi et al., 2019; Doan et al., 2021). Therefore, this study aimed to verify whether preoperative two-channel prefrontal EEG can be used to predict POD in elderly patients undergoing spinal surgery. We hypothesized that POD is associated with resting-state EEG biomarkers, as previously reported (Choi et al., 2019).

## 2. Materials and methods

### 2.1. Data collection

The study protocol was approved by the local institutional review board (Severance hospital 4-2019-0654, Clini-calTrials.gov Identifier: NCT04120272). All the patients provided written informed consent. Patients aged ≥ 70 years who were scheduled to receive general anesthesia for >2 h were considered eligible for this study. We excluded patients who were diagnosed with cognitive impairment or with a preoperative MMSE score of less than 23 from the study.

Patient characteristics, comorbidities, and social history were collected through patient interviews and medical chart reviews. Baseline cognitive function was examined using the MMSE and Montreal cognitive assessment (MoCA). Further, we collected patient characteristics [frailty index (Chen and Qin, 2021), global deterioration scale (Seo et al., 2014), instrumental activities of daily living (IADL) (Tognoni et al., 2011), and mini nutritional assessment (MNA) scores (Chu et al., 2016)] and co-morbidities [Charlson comorbidity index (CCI)]. Additionally, we collected intraoperative data (estimated blood loss, total anesthesia duration, and anesthetics). We postoperatively used the CAM to assess POD at least four times a day during the hospitalization period. Patients diagnosed with delirium using the CAM were evaluated for the duration of symptoms of delirium (e.g., reduced awareness of the environment; poor cognitive skills; behavioral changes, including hallucinations, restlessness, calling out, slowed movement, or lethargy; and emotional disturbances, such as anxiety, irritability, euphoria, apathy, unpredictable mood shift, and personality changes). Data on the POD subtype and cognitive impairment severity using the Korean version of the Delirium Rating Scale were collected. Postoperative pain was recorded and we recorded the daily consumption of opioid drugs for pain control.

**FIGURE 1:**
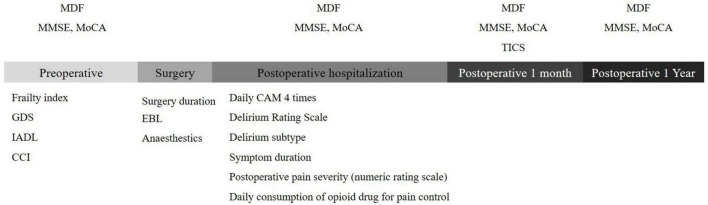
Sequential timeline of cognitive assessments. MDF, median dominant frequency; MoCA, montreal cognitive assessment; MMSE, mini-mental state examination; GDS, global deterioration scale; IADL, instrumental activities of daily living; CCI, charlson comorbidity index; EBL, estimated blood loss; TICS, telephone interview for cognitive status™.

Regarding postoperative follow-up examination of cognitive function, we measured the MMSE and MoCA scores on the 5th day after surgery. Due to patients were reluctant or unable to perform face-to-face follow-up inter-views due to COVID-19, long term follow-up cognitive function was examined using the telephone interview for cognitive status™ (TICS) after hospital discharge (Figure 1). The TICS is a brief standardized test of cognitive function that was developed for situations where in-person cognitive screening is impractical or inefficient (Robinson et al., 2021).

We assumed a Postoperative Delirium (POD) incidence rate of 25% in elderly patients, based on previous studies (Watne et al., 2014; Kim et al., 2016; Van Grootven et al., 2016). We expected the effect size (odds ratio) of MDF for the POD variable to be around 0.45. Statistically, it was determined that at least 207 subjects should be observed when calculating the sample size, at a significance level of 0.05 (alpha), ensuring a statistical power of 80%.

### 2.2. EEG acquisition and processing

Trained personnel performed EEG on patients seated in an upright position at the anesthesia consultation clinic between the hours of 2 p.m. and 5 p.m. Resting-state EEG was measured with the patients’ eyes closed using frontal electrodes for 5 min and was repeated at 5 days and 1 month after surgery. During the test, the participants were comfortably sat in a chair with regular lighting conditions. Non-invasive monopolar scalp electrodes were used to record electrical brain activity at two prefrontal regions (Fp1 and Fp2 in International 10/20 electrodes system), with a reference electrode on the right earlobe. The band-pass frequency of the neuroNicle amplifiers (LAXTHA Inc., South Korea) was 3–43 Hz; additionally, the input range was ± 393 μV (input noise < 0.6 μVrms). We applied the following filters (digital, Butterworth IIR) to the signal: band stop- 2nd order with f1 = 55 Hz and f2 = 65 Hz; high-pass- 1st order with fc = 2.6 Hz; and low-pass- 8th order with fc = 43 Hz. Contact impedance was maintained < 10 kΩ. All data were digitized in the continuous recording mode (5 min of EEG; 250 Hz sampling rate; 15-bit resolution).

We examined for data contamination in the (Fp1, Fp2)-prefrontal EEG signals caused by muscle and eye movement since no artifacts were rejected during signal processing. First, we confirmed that the EEG data was not contaminated by large artifacts [>10% of epochs exceeding a maximum amplitude of 200 μV (Noh et al., 2006)]. Further, we did not identify any artifacts after applying a stricter threshold (10% of epochs exceeding a maximum amplitude of 100 μV).

### 2.3. EEG metrics and computation

Electroencephalography of the eye-closed resting state used in our study is known to show a pattern with a very dominant intrinsic oscillation throughout the cerebral cortex (Choi et al., 2020). In terms of spectrum distribution, the pattern appears to have a high rise cantered on a specific frequency in the 5–12 Hz region where theta (4–8 Hz) and alpha wave (8–13 Hz) bands are simultaneously involved. Moreover, in older patients, the subjects of our study, the central frequency of this bell-shaped spectral distribution is relatively slower than that observed in young patients; therefore, the contribution of the rhythms spanning the theta and alpha boundaries is rather high, making conventional EEG indices based on theta and alpha power unsuitable for quantifying the delicate features of intrinsic oscillation that span the boundary between two rhythms as their frequency bands are artificially separated by fixed values for all subjects. A peak frequency (PF) index, which is determined by the frequency of the highest peak point, is sometimes introduced to characterize the representative frequency of intrinsic oscillation. However, it is difficult to obtain a consistent value in the case of a double peak shape in the spectral distribution or a slightly spread-out sideways peak shape. In recent studies, the introduction of the central frequency known as median dominant frequency (MDF) (Supplementary Figure 2), which corresponds to half the area in the bell-shaped spectral distribution, has provided more significant features than the existing alpha-to-theta ratio or PF ratio (Choi et al., 2019, 2020; Yi et al., 2019; Park et al., 2020; Doan et al., 2021). Therefore, the MDF was selected for this study, which requires the observation of delicate structural changes in intrinsic oscillation related to POD.

### 2.4. Statistical analyses and model construction

Continuous variables are expressed as mean ± standard deviation or median (interquartile range) and were compared using the independent *t*-test or Mann–Whitney U test depending on the data distribution. Categorical variables are expressed as numbers (%) and were compared using the chi-square test or Fisher’s exact test. Risk factors for delirium were identified through univariable logistic regression analysis. Since the multivariable logistic models applied the rule of ten events per variable, the variable of the multivariable logistic model was selected based on its clinical relevance among the risk factors in the univariable logistic result. Only the variables that showed significant effects in the univariable logistic regression analysis were included in the multivariable logistic regression analysis. Known delirium risk factors, which are considered clinically important factors for POD occurrence, were included in the model, and AIC (Akaike information criterion) values for all combinations of variables has been calculated. As a result, final model was selected with preoperative variables in univariable logistic regression The area under the receiver operating characteristic curve (AUC), calibration was performed to identify the agreement between the observed outcomes and predicted probabilities of postoperative delirium. Between-group comparisons of changes in the numeric rating scale score for pain and postoperative opioid dosage during the 7 postoperative days were performed using a linear mixed-effect model with compound symmetry covariance structure. Statistical analyses were performed using SAS v9.4 (SAS Institute Inc.) and R v4.2.2.^1^

## 3. Results

**FIGURE 2:**
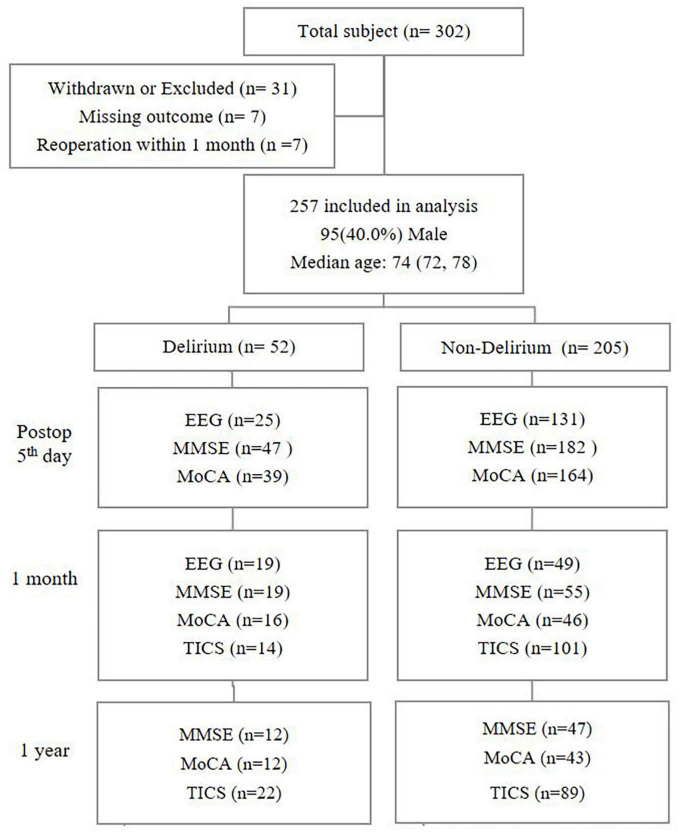
Summary of participant enrolment. The median age is shown with 25th and 75th percentiles.

Figure 2 summarizes the patient enrolment. We had assessed postoperative delirium in 257 patients until discharge; among them, 52 (20.2%) had delirium.

Table 1 presents the preoperative scores for various measures.

**TABLE 1:** Participant characteristics.

	Delirium (*n* = 52)	Non-delirium (*n* = 205)	*P*-value
Age (years)	76 (73, 79)	74 (71, 77)	0.002*
Sex			0.129
Men	14 (26.9%)	81 (39.5%)	
Women	38 (73.1%)	124 (60.5%)	
MDF (Median Dominant Frequency)	8.65 ± 0.70	9.04 ± 0.59	<0.001*
MMSE (Mini-Mental State Examination)	27 (25, 28)	27 (26, 29)	0.076
MoCA (Montreal Cognitive Assessment)	22 (19, 25)	24 (21, 26)	0.039*
GDS (Global deterioration scale)	4 (2, 7)	3 (1, 7)	0.687
IADL (Instrumental activities of daily living)	11 (10, 16)	10 (10, 13)	0.009*
Frailty index	2 (1.75, 3)	2 (1, 3)	0.025*
Mini nutritional assessment	13 (11, 14)	14 (13, 15)	0.001*
CCI (Charlson comorbidity index)	4.5 (4, 5)	4 (3, 4)	<0.001*
DRS (Delirium Rating Scale)	22.3 ± 8.8		

For continuous variables, those presented as mean ± standard deviation were analyzed using the independent *t*-test, while variables expressed as median (25th–75th percentile) were evaluated using the Mann–Whitney U test. For categorical variables, the chi-square test was performed. *p < 0.05 compared with non-delirium group.

Compared with patients without POD, those with POD were older and had lower MDF, lower MOCA, lower daily activities, poor nutrition state, higher frailty, and higher CCI score. The median duration of POD was 2 (1, 3.25) days, while the average Delirium Rating Scale score was 22.3 points (Table 1). POD usually occurs within 72 h after surgery, but it can also occur on the fifth day after surgery, and symptoms may remain a week after surgery. Among the 52 patients with delirium, 22, 24, and 6 were classified as having the hyperactive, mixed, and hypoactive types, respectively.

### 3.1. Univariable analysis

Univariable analysis by logistic regression revealed that numerous preoperative factors and patient characteristics were associated with an increased risk of developing POD. These results are shown in Table 2. Notably, the MDF was associated with decreased odds of POD; specifically, a 1-Hz decrease in the MDF increased the odds for developing POD by 2.78 times. Notable patient characteristics with odds ratios (OR) were age, IADL, Frailty index and a CCI score.

**TABLE 2:** Univariable logistic regression for postoperative delirium.

	Crude OR (95% CI)	*P*-value
Age	1.13 (1.05–1.23)	0.001*
Sex		
Men	1 (ref)	
Women	1.77 (0.92–3.58)	0.100
Preoperative MDF (Median Dominant Frequency)	0.36 (0.21–0.60)	<0.001*
Preoperative MMSE (Mini-Mental State Examination)	0.88 (0.78–1.00)	0.050
Preoperative MoCA (Montreal Cognitive Assessment)	0.91 (0.84–0.99)	0.024*
GDS (Global deterioration scale)	1.01 (0.93–1.09)	0.900
IADL (Instrumental activities of daily living)	1.09 (1.01–1.18)	0.032*
Frailty index	1.43 (1.08–1.91)	0.014*
Mini nutritional assessment	0.82 (0.72–0.93)	0.001*
CCI (Charlson comorbidity index)	1.75 (1.33–2.34)	<0.001*

*p < 0.05 compared with the non-delirium group.

### 3.2. Multivariable analysis

**FIGURE 3:**
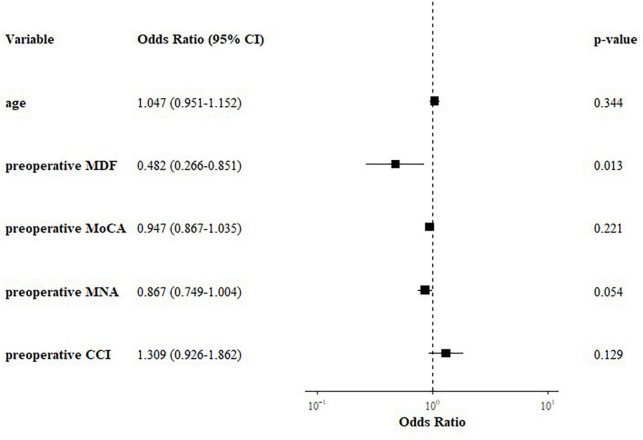
Forest plot of multivariable logistic regression results for postoperative delirium.

A multivariable logistic regression analysis that included age, MDF, MoCA score, MNA, and CCI revealed that only preoperative MDF was significant (Odds ratio = 0.48, 95% confidence interval 0.27–0.85, *p* = 0.013) (Figure 3).

**FIGURE 4:**
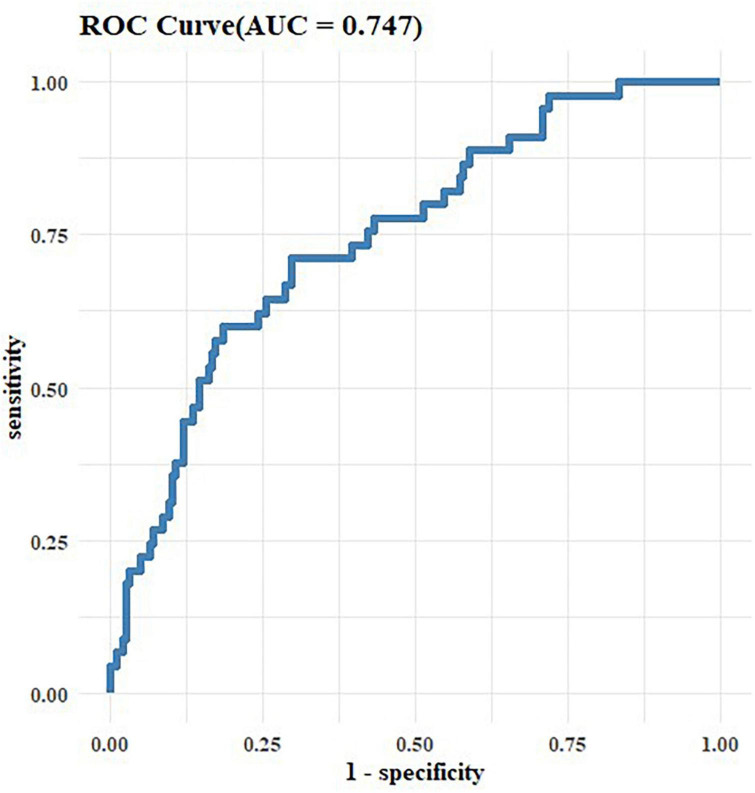
Receiver operating characteristic (ROC) of regression model.

We assessed the goodness-of-fit of the logistic regression model using the Hosmer-Lemeshow test, which yielded a test statistic of 6.344 and a *p*-value of 0.61. The area under the curve (AUC) of the prediction model comprising the age, MDF, MoCA score, MNA, and CCI score was 0.747 (Figure 4).

### 3.3. Postoperative long-term outcome

**FIGURE 5:**
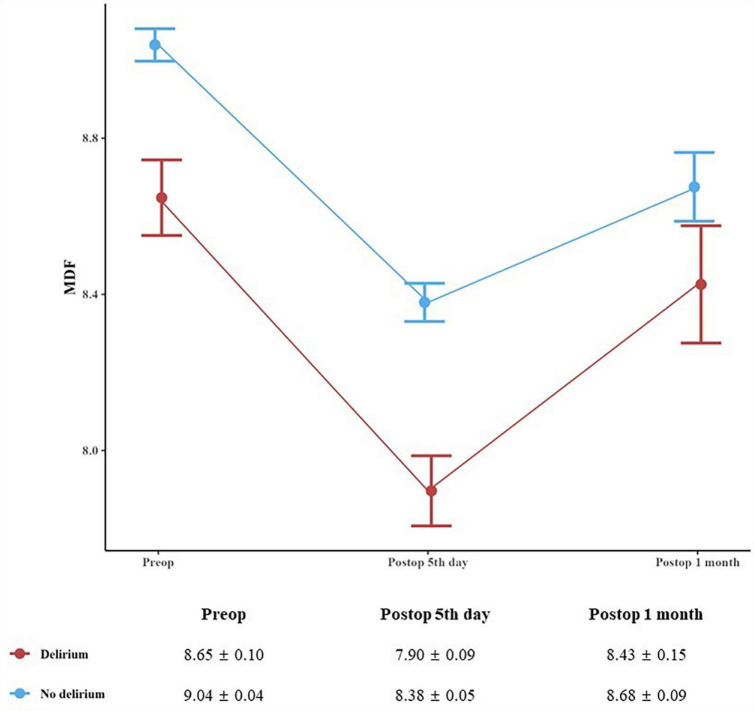
Median dominant frequency before surgery as well as 5 days, and 1 month after surgery. A linear mixed model with compound symmetry covariance structure interaction effect of group × time adjusted for age revealed a significant result (*p* for difference = 0.001, *p* for interaction = 0.038). Data are presented as the geometric mean ± the standard error of the mean.

Compared with patients without POD, those with POD showed a significantly longer median duration of postoperative hospitalization by Mann–Whitney U test (11 days vs. 9 days, *p* = 0.001). However, there was no significant between-group difference in the ratio of patients who returned home after hospital discharge to those who were transferred to other facilities for rehabilitation purposes (data not shown). Using a linear mixed model and adjusting for age, we found that in the delirium group, there was a significant difference in MDF values between groups (*p* = 0.001), as well as a significant interaction between MDF and time points (*p* = 0.038). The difference in MDF between groups persisted from before surgery to 1 month after surgery (Figure 5). To address the discrepancies in follow-up EEG test rates: Patients with postoperative delirium, informed about delirium and EEGs, were more inclined to seek further tests. Additionally, those not undergoing follow-up MDF evaluations showed no clear biases in preoperative MDF values, suggesting that the follow-up data is likely representative of the overall group.

Compared with the non-delirium group, the delirium group showed non-significantly lower MMSE and MoCA scores during the immediate postoperative period. And patients with delirium showed significantly lower TICS scores until 1 postoperative year after hospital discharge (Supplementary Table 1). Based TICS scores, cognitive impairment in patients with POD could lasted up to 1 postoperative year. For postoperative pain severity and opioid consumption, the delirium group showed persistent pain on resting (Supplementary Figure 1) although there were no between-group differences in the analgesic doses (data not shown).

## 4. Discussion

This prospective study revealed a relationship between preoperative median dominant frequency (MDF) with postoperative delirium (POD). Specifically, lower MDF values could moderately predict POD occurrence. Additionally, patients with POD showed a sharp decrease in the MDF on the 5th postoperative day, which persisted until 1 month after surgery. Follow-up TICS measurements suggest that the cognitive dysfunction might persist until 1 year after surgery.

There have been recent studies on the predictive utility of EEG for dementia. Alpha power is positively correlated with hippocampal atrophy and cognitive function (Luckhaus et al., 2008). Compared with other modalities, EEG can promptly detect subtle changes in brain function, especially early-stage cognitive decline. A previous study report that prefrontal EEG markers outperform MMSE scores in predicting dementia, combining both measures can yield more prediction accuracy (Doan et al., 2021). Therefore, EEG has wide potential applications as a screening tool for cognitive impairment, including POD prediction. However, to our knowledge, there have been no studies on the predictive utility of preoperative EEG for POD.

Previous studies have demonstrated a negative correlation between prefrontal EEG biomarker, MDF and brain aging in healthy participants, with the resting-state eyes-closed MDF in younger and older adults being >10 Hz and <9 Hz, respectively (Choi et al., 2020). Further, in the elderly population, the prefrontal MDF was positively correlated with the MMSE scores. The mean value of alpha oscillation in patients with MCI and dementia is approximately 8.3 Hz (Choi et al., 2019) and 6.6 Hz (Doan et al., 2021), respectively. We observed a significant between-group difference in the MDF [delirium (8.65 Hz) vs. non-delirium (9.04 Hz)]. MDF is a neural network system that is active at resting status and is well known as Default Mode Network (DMN). The rhythm of this DMN is intrinsic oscillation, and the functional and anatomical disconnection of DMN could represent a core feature of mild cognitive impairment and postoperative cognitive dysfunction (Berger et al., 2019). Recent study has shown that decreasing of MDF was associated with the pre-dementia stage of Alzheimer’s disease (Choi et al., 2023).

We only included patients with normal cognitive function at baseline based on the MMSE score. However, the delirium group showed a lower preoperative MDF than the non-delirium group, which suggests that the patients with delirium could have had preoperative undiagnosed MCI. This is further indicated by our MoCA findings. The MMSE (Folstein et al., 1975) is a quick and easy measure of cognitive functioning that has been widely used in clinical and research evaluation of patients with dementia. However, it has a relatively low sensitivity for MCI, with increased false-negative rates. Contrastingly, the MoCA was designed to screen for both MCI and dementia (Larner, 2012; Pinto et al., 2019). The preoperative MoCA scores in our patients with delirium were consistent with those in previously reported patients with MCI [average MoCA scores for MCI and mild AD were 22 (IQR, 19–25) and 16 (IQR, 11–21), respectively] (Nasreddine et al., 2005). Furthermore, the median preoperative MoCA score in the delirium group in our study was 22 points, which suggests that the delirium group could have had preoperative MCI.

In addition, there are reports that the great physical stress of surgery can affect patients’ EEG. According to a recent study by Labonte et al. (2023) young volunteers exposed to inhalation anesthetics demonstrated notable EEG changes. Building on these findings, our study investigated the connection between prefrontal EEG biomarkers and postoperative delirium in the elderly. Collectively, the research underscores the pivotal role of EEG metrics in understanding postoperative cognitive dysfunction, especially in vulnerable patient.

On the 5th day after surgery, the MDF of the delirium group was especially lower than that previously reported in patients with MCI (Choi et al., 2019). By linear mixed model analysis, it was found that this difference showed a significant difference until 1 month after surgery (Figure 5). Due to COVID-19 phobia, the decrease of follow-up postoperative hospital visits has affected our research design. In particular, elderly patients became reluctant to face-to-face tests, so they preferred cognitive tests over the phone rather than inter-view-based tests that required dialogue between subjects and researchers such as MMSE and MoCA. At the 1-month and 1-year postoperative follow-ups, the delirium group showed lower TICS scores than the non-delirium group (Supplementary Table 1). This is consistent with previous reports of reduced TICS scores in patients with MCI than in normal controls (32.81 and 37.28, respectively) (Salazar et al., 2014).

Postoperative delirium is associated with several adverse clinical effects, including dementia, distress, increased hospitalization du-ration and costs, and higher mortality (Bellelli et al., 2014). In line with previous studies, we observed significant between-group differences in the IADL, frailty index, MNA, and CCI scores.

This study had some limitations. The main limitation of this study is that TICS is used as a long-term follow up test. Although, in elderly population, the TICS performed well as a diagnostic screening tool for excluding dementia and it is particularly useful when face-to-face diagnostic screening is not feasible (Abdulrahman et al., 2022). Long-term follow-up test results would have been statistically more clear if we performed the same test used during perioperative period. Second, various EEG parameters that are known early markers for the prognosis of MCI or dementia were not analyzed. Our study analyzed only a single indicator, MDF, other than parameters such as the power ratio of α3/α2 or θ/α1 ratio related to degenerative cognitive disorders. For a more explicit prediction of POD, a further study with additional EEG paradigms of event-related potential related to surgery would be useful. Nevertheless, a recent study revealed that eye-closed resting-state EEG features, MDF could be a good indicator of neuro-degeneration and which is advantageous to practicality in real clinical field (Choi et al., 2023). Notwithstanding these limitations, the study suggests that the preoperative MDF is related to POD. Conducting a comprehensive investigation with a larger sample size would enable a more robust analysis of the relationship between preoperative MDF and POD. Additionally, it would provide an opportunity to explore targeted interventions based on EEG patterns to mitigate the occurrence and severity of POD. In conclusion, a low MDF on preoperative resting state prefrontal EEG was associated with POD in elderly patients undergoing spinal surgery. Our findings demonstrate the EEG can serve as potential marker for identifying patients at a high risk of POD.

## Data availability statement

The original contributions presented in this study are included in the article/Supplementary material, further inquiries can be directed to the corresponding authors.

## Ethics statement

The studies involving humans were approved by Severance Hospital 4-2019-0654. The studies were conducted in accordance with the local legislation and institutional requirements. The participants provided their written informed consent to participate in this study.

## Author contributions

JK helped in the study design, data acquisition, data analysis and interpretation, statistical analysis, manuscript drafting, and approval of the submitted version of the manuscript. SP and WC helped in the data analysis and interpretation, manuscript drafting, and approval of the submitted version of the manuscript. K-NK and YH helped in the data acquisition, data analysis, and approval of the submitted version of the manuscript. S-JS helped in the statistical analysis and interpretation, manuscript drafting, and approval of the submitted version of the manuscript. K-YL, JC, and B-NK helped in the study design, data analysis and interpretation, statistical analysis, manuscript drafting, and approval of the submitted version of the manuscript. All authors contributed to the article and approved the submitted version.

## Footnotes

1
http://www.r-project.org/
