# Supplementary material for: Resting-state prefrontal EEG biomarker in correlation with postoperative delirium in elderly patients
Source: Front Aging Neurosci. 2023 Sep 25;15:1224264. doi: 10.3389/fnagi.2023.1224264 (PMC10561289; doi:10.3389/fnagi.2023.1224264)
Supplement: Supplementary file 1 [file Data_Sheet_1.docx]

Supplementary Material

Resting-state prefrontal EEG biomarker in correlation with

postoperative delirium in elderly

patients Jeongmin Kim^1,2^, Sujung Park^1,2^, Keung-Nyun Kim^3^, Yoon Ha^3,4^, Sang-Jun Shin^5^, Wonseok Cha^6^, and Ki-young Lee^1,2*†^, Jungmi Choi^6 *†^, Bon-Nyeo Koo^1,2*†^

^1^Department of Anesthesiology and Pain Medicine, Yonsei University College of Medicine, Seoul, Republic of Korea.

^2^Anesthesia and Pain Research Institute, Yonsei University College of Medicine, Seoul, Republic of Korea.

^3^Department of Neurosurgery, Spine and Spinal Cord Institute, Severance Hospital, Yonsei University College of Medicine, Seoul, Korea.

^4^POSTECH Biotech Center, Pohang University of Science and Technology, Pohang, Korea.

^5^Department of Biomedical Systems Informatics, Biostatistics Collaboration Unit, Yonsei University College of Medicine, Seoul, Republic of Korea.

^6^Human Anti-Aging Standards Research Institute, Gyeongsangnam-do, Republic of Korea

**
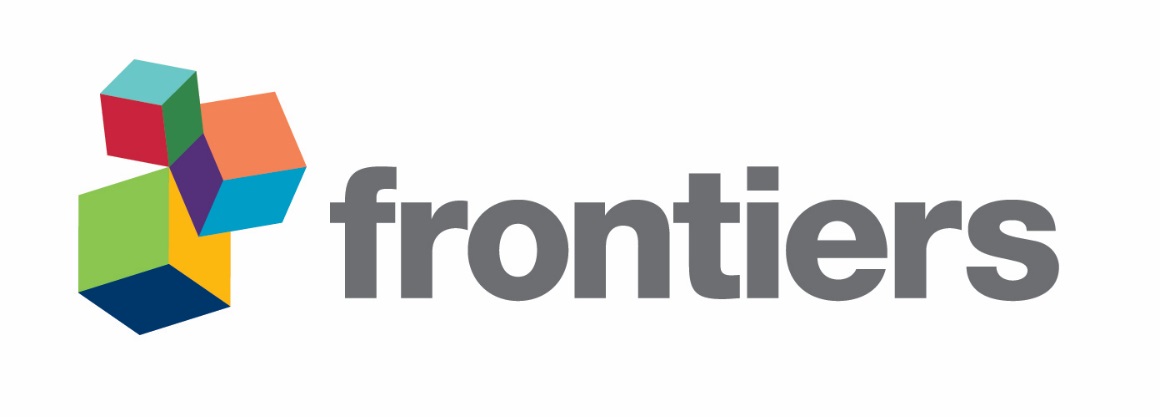
**

**Supplementary Figure 1.** Numeric rating scale (NRS) score for postoperative pain. Linear mixed model with compound symmetry covariance structure revealed a significant interaction effect of group*time (p = 0.002) For the covariance structure, unstructured covariance was used considering the AIC (Akaike Information Criterion) value.


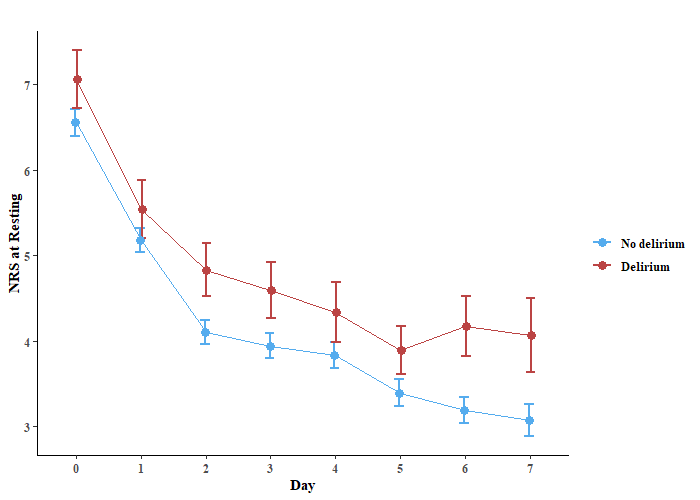


**Supplementary Figure 2. Calculated MDF markers for Non-delirium and Delirium groups**


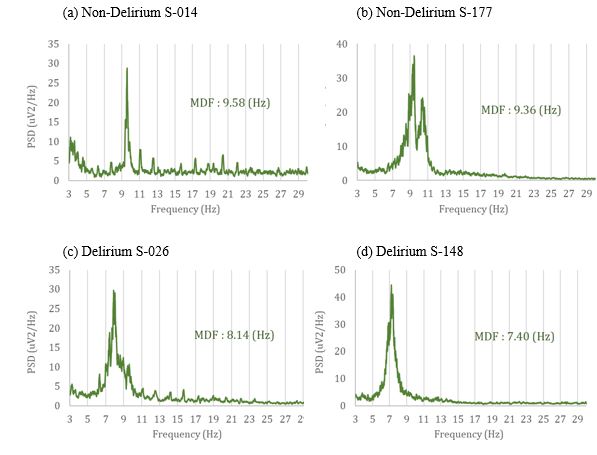


**Supplementary Table 1:** Cognitive function tests and EEG measurements performed before surgery and on day 5, 1 month, and 1 year after surgery

| Time | Test | Patient  number (%) | Delirium  (n= 52) | Non-Delirium  (n= 205) | *p* value |
| --- | --- | --- | --- | --- | --- |
| Preoperative | MMSE | n (%) | 52 (100) | 205 (100) | 0.076 |
|  |  |  | 27 [25;28] | 27 [26;29] |  |
|  | MoCA | n (%) | 45 (86.5) | 189 (92.2) | 0.0039* |
|  |  |  | 22 [19;25] | 24 [21;26] |  |
|  | MDF | n (%) | 52 (100) | 200 (97.6) | <0.001* |
|  |  |  | 8.65 ± 0.70 | 9.04 ± 0.59 |  |
| 5^th^ postoperative  day | MMSE | n (%) | 47 (90.4) | 182 (88.8) | <0.001* |
|  |  |  | 24 [21;27] | 27.5 [25;29] |  |
|  | MoCA | n (%) | 39 (75.0) | 164 (80.0) | <0.001* |
|  |  |  | 18 [14.5;24.5] | 23.5 [20;26] |  |
|  | MDF | n (%) | 25 (48.1) | 131 (63.9) | <0.001* |
|  |  |  | 7.90 ± 0.45 | 8.38 ± 0.56 |  |
| 1 postoperative  month | TICs | n (%) | 14 (26.9) | 101 (49.3) | 0.001* |
|  |  |  | 27.6 ± 7.3 | 33.4 ± 5.5 |  |
|  | MDF | n (%) | 19 (36.5) | 49 (23.9) | 0.192 |
|  |  |  | 8.58 [8.02;8.88] | 8.62 [8.31;9.23] |  |
| 1 postoperative  year | TICs | n (%) | 22 (42.3) | 89 (42.0) | 0.011* |
|  |  |  | 31.82 ± 6.62 | 35.72 ± 6.24 |  |

MMSE, Mini-mental State Exam; MoCA, Montreal Cognitive Assessment; TICS, Telephone Interview for Cognitive Status™; MDF, median dominant frequency, Numbers are median (25^th^–75^th^ percentile) or mean ± standard deviation *; *p* < 0.05 compared with non-delirium group
